# Some Conditions of Medical Progress

**Published:** 1892-03-19

**Authors:** 


### The Hospital

A Weekly Journal of

### Science, Medicine, Nursing, and Philanthropy.

*For Hospitals, Asylums, Infirmaries, Dispensaries, Medical Practitioners, Students, Nurses, and the Charitable Public.*

Vol. XI.—No. 286.] [March 19, 1892.

### Some Conditions of Medical Progress.

Certain critics have said of Lord Byron that he was a much greater poet than he ever, by his works, proved himself to be. To which it has been pertinently replied that he would have been a greater poet if he had produced greater works. This is to take up the position that a man is to be judged by what he does, rather than by what he is. Without attempting to argue the point, one cannot but see that the contention has at least the merit of plausibility. The world as a whole can never judge a man by what he is, because only the most infinitesimal fraction of its people can see and know any man personally, and even those who do see and know him do not by any means always know him as he is, but only as he seems to be to them. It must, therefore, ordinarily be the surer way of arriving at a just estimation of the value of a man to judge him by what he does, rather than by what he is, or appears to those who know him to be.

Medicine in all its departments, both scientific and practical, is to be judged in this way, and in no other. The surgeons, as distinguished from the physicians of the present time, have risen very high in the public estimation, and, perhaps, it may be hinted, in their own. To this no proper objection can be taken. They are judged by their works, and so judged they come out well. They have, with boldness and intelligence, entered upon new theories and new operations, and their boldness has been justified by results. Ovariotomy is now an old story, and cranial surgery bids fair to be a still more distinguished rival.

But, when all is said, it will be admitted that surgery can never cover the whole ground of medical practice. It promises to encroach more and more, and some of its methods are wisely adopted by physicians. But when it has gained all the ground which should of scientific right belong to it, there will still be left, for the practice of medicine pure and simple, a widely extended field. It is obvious, therefore, that the proper line of things for physicians is not one of humble ancillarity to surgeons, but one of cautious and conservative boldness in pushing their own researches and practice into every region warranted by a legitimate science. There is indeed no lack of boldness on the part of those who, whilst they would not perhaps call themselves physicians, would yet admit that their work, if it is to be serviceable at all to practical medicine, must be applied, in its widest extent, by those who use the medical rather than the surgical art. That boldness is eminently necessary and wise. The bacteriologists, and the experimenters of all classes, have entered upon new regions which it behoves mankind by all means to conquer. We cannot with patience sit down where we are. The idea of finality is intolerable to any man who takes anything like a just measure of past and present medicine. Practising physicians know something that is useful to man; the best of them indeed know a very great deal. Some few may be described as encyc[illegible]edias of diverse medical knowledge, and as the very acutest of judges in its rational application to practice. But even those who know so much, and use their knowledge so well, would be the first to say that they look for a time when the profundities of to-day will be the common-places of to-morrow, and the physiological secrets which now baffle experts will be mere rudiments to future generations.

What one feels, and feels most painfully, is that knowledge which can be used at the bedside is conquered with exceeding slowness. The most grievous fact in this connection is that many physiological workers appear to despise the bedside. But what then is medicine for? And what is the object of all our physiological researches, and our pryings into the secret places where diseases have their origins? It is not difficult to understand that a bacteriologist who has gone down into the dark regions where micro-organisms live their lives and display their miraculous activities, should become fascinated by the new and unlooked for marvels which frequently reward his search. But even the bacteriologist, being a man of science, must feel the want of some criterion whereby to test, not only the soundness of his conclusions, but the practical value of his work. Such a criterion the bedside, and the bedside alone, can give. This may seem a little hard upon the bacteriologist, who is inclined to despise, if not the bedside, at least that doubtfully scientific hierarchy which presides over its medical mysteries.

It is no mark of wisdom, or of scientific breadth and justice, to despise either the bedside or those who elect to find their life-work there. No useful purpose would be served by contrasting the practising physician with the laboratory physiologist, to the exaltation or the abasement of either. But this, at least, the physiologist may understand—that the practising physician follows the work of the laboratory with eager and sympathetic eye. It is to it that he looks, as he stands in the thick of an increasingly arduous conflict, to supply him with weapons that shall not disappoint his hopes by their feebleness, but shall develop in his heart a strength and a certitude that shall pluck victory even out of the jaws of defeat.

What more striking proof of the intense interest which centres about the physiological laboratory could have been furnished than the spectacle of all the medical world rushing to Berlin to behold the wonders discovered by Koch? Does anyone suppose that a single medical man in Great Britain was disposed to judge Koch hardly because he was a German and not an English doctor? So far from that being so, every man among us was ready to give almost his right hand if Koch's tuberculine might have but proved a "cure" which actually "cured" and made people well.

The medical world criticised Koch, as a matter of course. Such criticism did not show the paucity, but the abundance of the interest which we all felt in him and his work. If tuberculine had but definitely and actually restored consumptives to health with reasonable frequency, Koch would have been held by the whole modern world of medicine to have been, not only the greatest physician of his time, but greater than the greatest of all who had preceded him. Does not this story of Koch show to physiologists the invaluable nature of the bedside as a touchstone and standard whereby all their work is to be tested? The primest of all the conditions of medical progress is that every supposed discovery shall triumphantly pass the examination which the homely bedside sets up. A hard condition, no doubt, for young and ardent workers, such as laboratory physiologists mostly are, but a condition which can by no possibility be dispensed with. Just as no young man can be entitled to the honour of a licence to practise medicine without first passing the imperative examinations, so can no discovery of the laboratory, and no method of cure elsewhere originated, ever receive the honour of general acceptance until it has passed the reasonable test of bedside utility.

Definiteness and actuality are the notes of modern medicine. Mere theories, however beautiful, and however supported by *a priori* reasonings, are entirely out of the running. Will the engine work? we ask. Will the remedy cure? If it will not, let us waste no more time in discussing it. Happily for the physiologists, and for modern medicine generally, we have a clinical laboratory where results can be ascertained with almost as much precision as the chemist or the bacteriologist can show. The well-regulated modern hospital is our place of therapeutic testing. The workers who conduct their researches there use all the methods of science, and control them by the temper of science. They have been trained to observe dispassionately and judicially, and to report exactly what they find. Any newly-discovered treatment which can run the gauntlet of all the hospitals, and come out alive at the end, is pretty sure of a long survival. Living as we do in a time when physiologists push forward their investigations with almost preternatural urgency, and bedside physicians, both in and out of hospitals, are equally intense in their desire to submit every discovery to the test of actual practice, it is impossible but that solid and enduring progress shall be made. That progress in medicine, like progress in religion, is often both halting and slow, we cannot deny. But slow or quick, halting or steady, it is certain that progress is being made, and that it promises to continue to be made in the future at a pace which will be constantly accelerated.

